# Shortened Daily Photoperiod Alleviates Anxiety-like Behaviour by Antioxidant Effect and Changes Serum Fatty Acid Profile in Diabetic Rats

**DOI:** 10.3390/jpm13050744

**Published:** 2023-04-27

**Authors:** Dolika D. Vasović, Milena Vesković, Nikola Šutulović, Dragan Hrnčić, Marija Takić, Đurđa Jerotić, Marija Matić, Olivera Stanojlović, Sanja Ivković, Irena Jovanović Macura, Dušan Mladenović

**Affiliations:** 1 Eye Hospital, University Clinical Centre of Serbia, 11000 Belgrade, Serbia; 2 Institute of Pathophysiology “Ljubodrag Buba Mihailovic”, Faculty of Medicine, University of Belgrade, 11000 Belgrade, Serbia; 3 Laboratory for Neurophysiology, Institute of Medical Physiology “Richard Burian”, Faculty of Medicine, University of Belgrade, 11000 Belgrade, Serbia; 4 Group for Nutrition and Metabolism, Centre of Research Excellence in Nutrition and Metabolism, Institute for Medical Research, University of Belgrade, 11000 Belgrade, Serbia; 5 Institute of Medical and Clinical Biochemistry, Faculty of Medicine, University of Belgrade, 11000 Belgrade, Serbia; 6 Department of Molecular Biology and Endocrinology, Vinca—Institute for Nuclear Sciences, National Institute of Republic of Serbia, University of Belgrade, 11000 Belgrade, Serbia; 7 Institute for Biological Research “Sinisa Stankovic”, National Institute of Republic of Serbia, University of Belgrade, 11000 Belgrade, Serbia

**Keywords:** shortened daily photoperiod, anxiety-like behaviour, oxidative stress, fatty acids profile, diabetes mellitus

## Abstract

The aim of our study was to investigate the effects of a shortened daily photoperiod on anxiety-like behaviour, brain oxidative stress, lipid status and fatty acid composition of serum lipids in a streptozotocin (STZ)-induced model of diabetes mellitus in rats. Male Wistar rats were divided into the following groups: first group—control group (C_12/12_); second group—diabetic group (DM_12/12_; 100 mg/kg STZ); third group—control group exposed to a light/dark cycle 6/18 h (C_6/18_); fourth group—diabetic group exposed to a light/dark cycle 6/18 h (DM_6/18_). Anxiety-like behaviour was tested three weeks following STZ injection by elevated plus maze (EPM) and open-field test (OFT). Oxidative stress parameters were measured in the cortex, hippocampus and thalamus, while lipid status and fatty acid methyl esters (FAMEs) were measured in the serum. Both EPM and OFT showed a lower degree of anxiety-like behaviour in the DM_6/18_ vs. DM_12/12_ group. Lipid peroxidation in the cortex, hippocampus and thalamus was significantly lower in the DM_6/18_ vs. DM_12/12_ group (*p* < 0.05), associated with an increased level of antioxidant enzymes and protein thiols in the cortex and thalamus. In the DM_6/18_ group, oleic, vaccenic, dihomo-γ-linolenic and docosahexaenoic acid concentrations were significantly higher in comparison to the DM_12/12_ group. A shortened daily photoperiod alleviates anxiety-like behaviour in diabetic rats by reduced lipid peroxidation and changes in the serum fatty acids profile.

## 1. Introduction

Diabetes mellitus (DM) is defined as a chronic metabolic disorder of multiple aetiology which is characterized by hyperglycaemia caused by inadequate insulin secretion, insulin action or both. Prolonged hyperglycaemia results in both microvascular and macrovascular complications leading to the development of retinopathy, neuropathy, nephropathy and cardiomyopathy, as well as accelerated atherogenesis [1]. Additionally, numerous studies have demonstrated increased prevalence of neuropsychiatric disorders such as depression, anxiety, and cognitive impairment in patients with diabetes [2,3].

The pathophysiological mechanisms of mood disorders in diabetic individuals remain unclear due to their complexity and the involvement of multiple factors. Although earlier studies emphasized the role of GABAergic and serotoninergic transmission disturbances in the pathogenesis of anxiety [4], recently the role of oxidative stress in the development of mood disorders has been clearly established [5]. Diabetes induces oxidative stress in the brain due to excessive glucose oxidation, non-enzymatic protein glycation and the subsequent oxidative breakdown of glycated proteins [6]. On the other side, anxiety- and depression-like behaviour may be ameliorated by antioxidants via up-regulation of the sirtuin 1 (SIRT1)–nuclear factor erythroid 2-related factor 2 (Nrf2)–haeme oxygenase 1 (HO-1)–glutathione peroxidase 4 (Gpx4) axis [7]. Curcumin and hydrogen sulphide also exert an anxiolytic effect via a reduction in oxidative stress and ferroptosis [8,9]. This clearly suggests that lifestyle measures that reduce oxidative stress may potentially alleviate diabetes-induced anxiety.

The role of fatty acids in DM-induced anxiety is less clear; however, much evidence suggests that fatty acids may modulate mood [10,11,12,13]. It is known that an increased level of fatty acids in DM is associated with metainflammation and neuroinflammation, contributing to anxiety-like behaviour [10]. Recent clinical trials confirmed that exogenous supplementation with n-3 and n-6 polyunsaturated fatty acids (PUFA) reduced serum inflammatory markers and anxiety in medical students [11], middle-age women [12] and in menopause [13]. Fatty acid level in the brain is closely interrelated to oxidative stress, since free fatty acids (FFAs) may be converted into toxic metabolites like ceramide and diacylglycerol, which stimulate ROS production [6].

Circadian misalignment may increase the risk of DM development, as well as exacerbate symptoms of anxiety-like behaviour and depression via the dysregulation of clock genes [14,15,16]. Clock gene products influence mood by the transcriptional regulation of serotoninergic, dopaminergic and glucocorticoid pathways [17]. The major regulator of clock gene expression is melatonin, a pineal gland hormone that reaches its maximum concentration in the dark [18]. Apart from clock gene dysregulation, reduced melatonin secretion in circadian misalignment may promote DM-induced anxiety via increased oxidative stress, inflammation and apoptosis in the brain [19,20,21]. Additionally, melatonin deficiency may increase the serum FFA level in high-fat-diet-fed animals [22], thus suggesting a role of dysregulated lipid metabolism in the pathogenesis of DM-induced anxiety.

Although the role of circadian misalignment in the pathogenesis of DM has been suggested, the possible effect of increased dark exposure as a lifestyle measure in the prevention of DM-induced anxiety is not clear. Of course, diabetic complications can be best prevented by appropriate glucoregulation. However, despite the progress in diabetes therapy, a significant number of patients still do not have adequate glucoregulation throughout their life and there is a need for additional preventive measures to reduce the risk of complications including anxiety and to improve life quality. Based on previous background information, it may be postulated that a shortened daily light exposure as a natural stimulus for melatonin synthesis may reduce the risk of anxiety via the antioxidative effect of melatonin and modulation of lipid metabolism. Therefore, the aim of our study was to investigate the effects of a shortened daily photoperiod on anxiety-like behaviour, brain oxidative stress, lipid status and serum fatty acid methyl ester (FAME) concentrations in a streptozotocin (STZ)-induced model of DM in rats.

## 2. Experimental Design

### 2.1. Experimental Animals

Experiments were carried out on male Wistar rats weighing 170–200 g which were bought from the Military Medical Academy Breeding Laboratories in Belgrade at the age of 8 weeks. All animals were maintained in separate transparent plastic cages (55 × 35 × 30 cm) under regular laboratory settings (temperature 22 ± 2 °C, humidity levels 50%, light/dark cycle 12/12 h with lights turned on at 09:00 a.m. before the experiment) with free access to regular laboratory animal chow and tap water. All experiments were carried out in compliance with the European Council Directive (2010/63/EU) and were approved by the Ethical Committee of the University of Belgrade (Permission No 9101/1).

A total of 39 rats were randomly divided into the following groups: (i) control group (C_12/12_; n = 8), a saline-treated group exposed to a light/dark cycle of 12/12 h; (ii) group with diabetes mellitus exposed to a light/dark cycle of 12/12 h (DM_12/12_; n = 11); (iii) saline-treated group on a light/dark cycle of 6/18 h (C_6/18_; n = 8); and (iv) group with diabetes exposed to a light/dark cycle of 6/18 h (DM_6/18_; n = 12). A single intraperitoneal injection of streptozotocin (100 mg/kg; Sigma-Aldrich, St. Louis, MO, USA) freshly diluted in sodium citrate buffer at pH 4.5 was administrated on day 1 of the experiment to induce diabetes in the DM_12/12_ and DM_6/18_ groups [23,24,25]. Animals were exposed to experimental light/dark cycles as previously described for 3 weeks starting on the day of streptozotocin administration [26]. The dark condition was defined as a breeding room brightness < 0.0004 cd/m^2^. After 7 days, the glucose concentration in the blood obtained from the tail vein was measured, and the rats with glycaemia >16.5 mmol/L were used in further experiments. Blood glucose levels were measured again weekly to confirm diabetic status.

**Figure 1:**
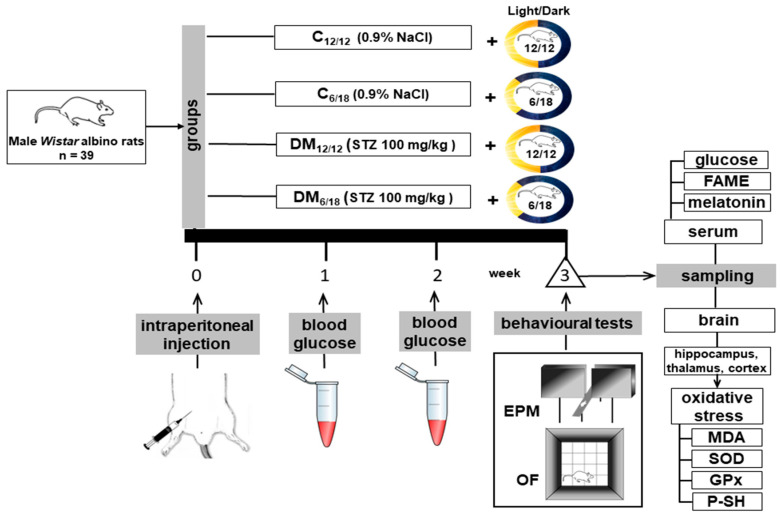
The schematic presentation of the experimental design. Abbreviations: MDA, malondialdehyde; SOD, superoxide dismutase; GPx, glutathione peroxidase; P-SH, protein thiol groups; STZ, streptozotocin; FAME, fatty acid methyl esters; EPM, elevated plus maze; OF, open field; DM_12/12_, diabetic group exposed to a 12/12 h light/dark cycle; C_6/18_, group exposed to a 6/18 h light/dark cycle; DM_6/18_, diabetic group exposed to a 6/18 h light/dark cycle.

The elevated plus maze (EPM) and open-field test (OFT) were used to assess anxiety-like behaviour three weeks after STZ injection. After behavioural testing, the blood was collected from the right side of the heart for biochemical analyses and the animals were sacrificed by decapitation. Blood samples were centrifuged for 15 min at 3000 rpm to separate serum for analysis of glucose, melatonin, FAME concentrations and lipid status. Brains were rapidly removed from the skull for oxidative stress analysis (Figure 1).

### 2.2. Behavioural Testing

The EPM test is recognized as one of the reliable tests for assessing anxiety-related behaviour in rodent models of CNS disorders [27]. It is composed of two open and two closed arms (50 × 10 cm) which are enclosed by 30-cm-high walls joined by a central platform. When placing rats on the central platform, the head was oriented towards the closed arm. In addition, rats were allowed to explore the maze for 5 min. Their movements and behaviour were recorded by a camera (HicVision Bullet 2612, Hangzhou City, Zhejiang, China). Behavioural parameters which were analysed included: (a) time which was spent in the open arms, (b) time which was spent in the closed arms, (c) the number of open-arm entries, and (d) the number of closed-arm entries. An arm entry was defined as the entry of all 4 limbs into that arm. Anxiety-like behaviour is characterized by a decrease in open-arm time, an increase in closed-arm time, a decrease in total transitions and an increase in closed-arm entries. The arms were cleaned with 70% ethanol and dried with paper towels after each trial. EPM testing was performed consistently at the end of the dark phase when animals were most active, under the bright lit conditions as described by Walf and Frye [28]. In the open-field apparatus, the OFT is used to assess both exploratory and locomotor activity. Since anxious animals spend more time on the periphery, the pattern of exploration can also be utilized as an indicator of anxiety. Exploratory activity was measured for 15 min immediately following EPM in a photo-cell-equipped activity box, as previously described in detail [29,30]. To monitor rat behaviour, an automated system equipped with infrared sensors (Experimetria Ltd., Budapest, Hungary) and its supporting software (Conducta 1.0) was used. The device tracks animals’ horizontal and vertical movement. A sound-proofed room with 12 lux red illumination was encircled by 40-cm-high black walls. The entire space was divided into 16 imaginary squares, with the centre portion defined by four middle squares. The time spent in the centre and the thigmotaxis index were recorded as indicators of anxiety-like behaviour. The thigmotaxis index was determined when the distance of rat ambulatory movements in the peripheral zones was divided by the total distance of ambulatory movements and expressed as a percentage. For the estimation of exploratory activity, the total ambulatory distance, distance travelled in the centre, ambulatory distance in the peripheral zone and the velocity of movements were measured. Anxiety was inversely related to the time spent in the central area and directly related to the thigmotaxis index.

Between trials, the open-field arena was cleaned with 70% ethanol.

### 2.3. Melatonin Determination

Melatonin concentrations were measured using a melatonin enzyme-linked immunosorbent assay (ELISA) kit (Abcam, ab213978, Cambridge, UK) in serum samples according to the manufacturer’s instructions. Each sample was run in duplicate.

### 2.4. Oxidative Stress

After brain removal from the skull, the cerebral cortex, hippocampus and thalamus were isolated on ice and stored at −70 °C before analysis. Lipid peroxidation, the activities of antioxidant enzymes and thiol groups were measured in these regions.

Lipid peroxidation was estimated based on the malondialdehyde (MDA) level using the competitive ELISA kit (Elabscience, Wuhan, China). The procedure was performed in accordance with the manufacturer’s instructions. Briefly, the method is based on the competition between MDA bound to the ELISA plate and MDA in the samples for biotinylated antibody. After the washing of unbound antibodies and the addition of HRP-conjugated secondary antibodies, the MDA level was determined spectrophotometrically at 450 nm.

Superoxide dismutase (SOD) activity was determined according to the Misra and Fridovich method based on the inhibition of epinephrine autooxidation by SOD [31]. Glutathione peroxidase (GPx) activity was estimated as reported by Günzler and Flohe [32]. Briefly, the activity of GPx is calculated based on the oxidation of NADPH with t-butyl-hydroperoxide as a substrate.

Protein thiol groups (P-SH) were determined based on the reduction of 5,5′-dithiobis-2-nitrobenzoic acid (DTNB) by P-SH which produces the yellow-coloured 5-thio-2-nitrobenzoic acid (TNB). This method was previously described by Jocelyn [33].

### 2.5. Lipid Status and FAME Determination

The concentrations of triglycerides and total and HDL-cholesterol concentrations were determined in serum using routine biochemical enzymatic methods described in detail elsewhere [34,35].

Blood samples’ (serum) lipids were trans-esterified to FAMEs with 3M hydrochloric acid in methanol according to a slightly modified procedure that has been reported by Manson and Waller [36] and Schlechtriem et al. [37]. Briefly, the butylated hydroxytoluene (10 mg/100 mL of methylation reagent, St. Louis, MO, USA) was added as an antioxidant. The reaction mixture containing plasma and methylation reagent was kept at 85 °C for 1 h and prepared samples were brought to ambient temperature in the next step. Then, they were mixed with hexane and centrifuged at 3000 rpm for 10 min. The upper phase was collected and dried under a gentle stream of N_2_. Finally, the obtained dry samples were reconstituted in hexane before analysis.

FAMEs were analysed by the gas–liquid chromatography method on GC-2014 gas chromatograph (Shimadzu, Kyoto, Japan) equipped with a flame ionization detector. The RTX 2330 column was 60-m long, having an internal diameter of 0.25 mm and a film thickness of 0.2 μm (RESTEK, Bellefonte, PA, USA), and was used for separation of the prepared plasma FAMEs samples. The column temperature setting has been described in detail elsewhere [36]. The commercially available fatty acids’ standards mixtures PUFA-2 and Supelco 37 Component Fame Mix (Supelco, Inc., Bellefonte, PA, USA) were used for the identification of individual FAMEs. The content of the individual fatty acids in plasma was calculated and expressed as a percentage of the total identified fatty acids in the lipid pool. The contents of the following 14 fatty acids were determined in the current study: the saturated fatty acids palmitic (C16:0) and stearic (C18:0) acid; monounsaturated palmitoleic (C16:1ω7), oleic (C18:1ω9) and vaccenic (C18:1ω7) acids; n-6 polyunsaturated linoleic (C18:2ω6), γ-linolenic (C18:3ω6), dihomo-γ-linolenic (C20:3ω6), arachidonic (C20:4ω6) and docosatetraenoic (C22:4ω6) acids; and n-3 polyunsaturated α-linolenic (C18:3ω3), eicosapentaenoic (EPA, C20:5ω3), docosapentaenoic (DPA, C22:5ω3) and docosahexaenoic (DHA, C22:6ω3) acids.

### 2.6. Statistical Analysis

Serum glucose, melatonin, FAMEs concentrations and lipid status were expressed as means ± SD. The EPM test results were presented as medians with 25th and 75th percentiles. The differences of OFT, biochemical and oxidative stress parameters were tested using one-way ANOVA followed by Dunnett’s post hoc test. Since the Kolmogorov–Smirnov test did not show a Gaussian distribution of the data, the significance of the differences in EPM test parameters was estimated by the nonparametric Kruskall–Wallis test with Dunnett’s post hoc test. The correlation between MDA in brain structures and anxiety-like behaviour, as well as between FAME concentrations and anxiety parameters, was analysed using Pearson and Spearman’s tests. Statistical analysis was performed using SPSS21.0 and GraphPad, and the differences and correlations were considered significant if *p* < 0.05.

## 3. Results

### 3.1. Blood Glucose and Melatonin Level

**Figure 2:**
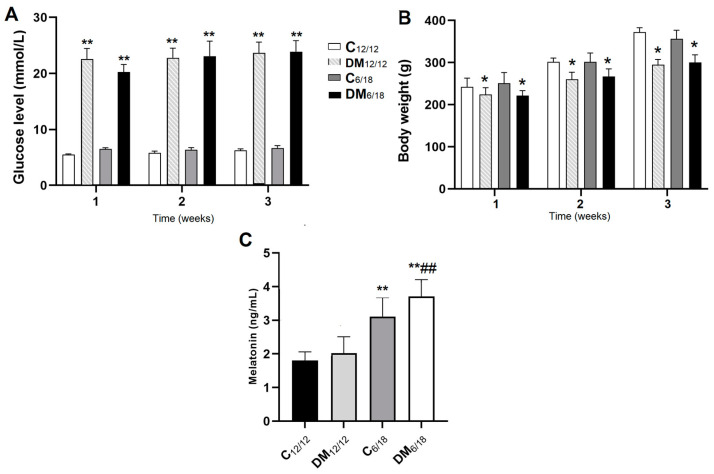
(**A**) Blood glucose, (**B**) body weight and (**C**) serum melatonin level in experimental animals. Diabetes was induced by a single intraperitoneal dose of streptozotocin (STZ, 100 mg/kg). Animals were subsequently exposed to a light/dark cycle of 6/18 h for 3 weeks (C_6/18_ and DM_6/18_ groups) or to a regular 12/12 h light/dark cycle (C_12/12_ and DM_12/12_ groups). Glucose was measured in blood samples obtained from the tail vein weekly within 3 weeks after STZ administration. Melatonin was determined in serum samples obtained from blood 3 weeks after STZ administration following behavioural testing. The significance of the difference was estimated by an analysis of variance (ANOVA) with Dunnett’s post hoc test (* *p* < 0.05 and ** *p* < 0.01 vs. C_12/12_, ## *p* < 0.01 vs. DM_12/12_). Abbreviations: DM_12/12_, diabetic group exposed to a 12/12 h light/dark cycle; C_6/18_, group exposed to a 6/18 h light/dark cycle; DM_6/18_, diabetic group exposed to a 6/18 h light/dark cycle.

The measurement of blood glucose level one week after STZ administration revealed that 9/11 animals from the DM_12/12_ group and 10/12 animals from the DM_6/18_ group developed hyperglycaemia. These animals were included for further analysis. The mortality rate was 2/9 in DM_12/12_ and 2/10 in DM_6/18_ until the end of the experimental protocol. The glucose levels were significantly higher in DM_12/12_ (22.5 ± 1.9 mmol/L) vs. both C_12/12_ (5.5 ± 0.1 mmol/L; *p* < 0.01) and C_6/18_ (6.5 ± 0.2 mmol/L; *p* < 0.01) at the end of the first week (Figure 2A). Significantly higher glucose levels were also observed in the DM_6/18_ group (20.2 ± 1.3 mmol/L) when compared to both the C_12/12_ and C_6/18_ groups (*p* < 0.01). There was no significant difference between the DM_6/18_ and DM_12/12_ groups (*p* > 0.05; Figure 2A). The body weight at the end of the first week was significantly lower in both the DM_12/12_ (221.11 ± 13.53 g) and DM_6/18_ (221.60 ± 12.61 g) groups when compared to the C_12/12_ (244.00 ± 14.33 g) and C_6/18_ (249.87 ± 15.87 g) groups (*p* < 0.05; Figure 2B). No significant difference between the C_12/12_ and C_6/18_ groups was observed (*p* > 0.05; Figure 2B). The same trends were observed at the end of the second and third week in blood glucose levels (*p* < 0.01; Figure 2A) and body weight (*p* < 0.05; Figure 2B).

The concentration of serum melatonin three weeks after STZ administration was significantly higher in both the C_6/18_ (3.24 ± 0.36 ng/mL) and DM_6/18_ groups (3.71 ± 0.22 ng/mL) when compared with the C_12/12_ (1.98 ± 0.26 ng/mL; *p* < 0.01) and DM_12/12_ groups (2.02 ± 0.22 ng/mL; *p* < 0.01; Figure 2C).

### 3.2. Behavioural Testing

**Figure 3:**
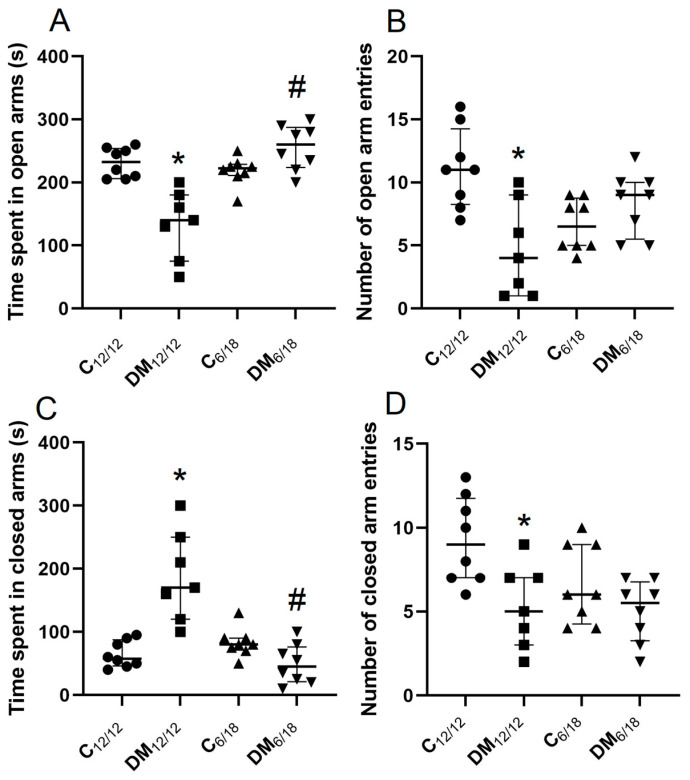
The effect of shortened daily photoperiod on elevated plus maze (EPM) parameters. The EPM consists of two open and two closed arms (50 × 10 cm) enclosed by 30-cm-high walls that are joined by a central platform. Rats were placed on the central platform with head oriented towards the closed arm and were allowed to explore the maze for 5 min. The parameters analysed in this test were: (**A**) time spent in the open arms, (**B**) number of open-arm entries, (**C**) time spent in the closed arms, (**D**) number of closed-arm entries. Anxiety-like behaviour is associated with a reduced time spent in the open arms, increased time spent in the closed arms and a reduced number of total transitions with an increased number of closed-arm entries. The significance of the difference was estimated by a Kruskall–Wallis test with Dunnett’s post hoc test (* *p* < 0.05 vs. C_12/12_, # *p* < 0.05 vs. DM_12/12_ group). ●∎▲▼ represent individual values in the C_12/12_, DM_12/12_, C_6/18_ and DM_6/18_ groups, respectively. For additional information, see Figure 2.

Anxiety-like behaviour in EPM is associated with a reduced time spent in the open arms and a reduced number of open-arm entries. The time spent in the open arms and the number of open-arm entries in EPM were significantly reduced in the DM_12/12_ vs. C_12/12_ group (*p* < 0.05, Figure 3A,B). However, the time spent in the open arms was significantly increased in DM_6/18_ by comparison with the DM_12/12_ group (*p* < 0.05, Figure 3A). The time spent in the open arms and the number of open arm entries were not significantly different between the DM_6/18_ and C_12/12_ groups (*p* > 0.05; Figure 3A,B), thus suggesting an anxiolytic effect of the shortened daily photoperiod. This anxiolytic effect was further confirmed by analysis of the time spent in the closed arms. The time spent in the closed arms was significantly higher in the DM_12/12_ vs. the C_12/12_ group (*p* < 0.05, Figure 3C), and significantly reduced in the DM_6/18_ vs. the DM_12/12_ group (*p* < 0.05; Figure 3C). In addition, the shortened daily photoperiod did not affect the number of closed-arm entries in diabetic animals, while it was significantly lower in the DM_12/12_ vs. the C_12/12_ group (*p* < 0.05; Figure 3D).

**Figure 4:**
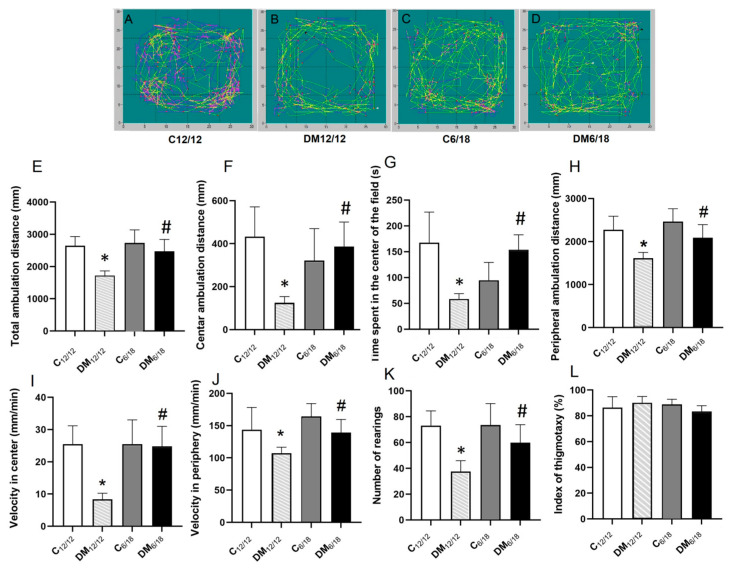
The effect of shortened daily photoperiod on open-field test (OFT) parameters. Horizontal and vertical motor activity of animals was registered for 15 min in a sound-attenuated area with 12 lux red lighting surrounded by black walls, 40-cm high. The whole area was divided into 16 imaginary squares with 4 middle squares marked as the central area. Representative images of animal ambulation are presented (**A**–**D**). Parameters obtained in this test included: (**E**) total ambulation distance, (**F**) centre ambulation distance, (**G**) time spent in the centre of the field, (**H**) peripheral ambulation distance, (**I**) velocity in the centre, (**J**) velocity in the periphery, (**K**) number of rearings, (**L**) index of thigmotaxy, defined as a ratio between the distance of rat ambulatory movements in the peripheral zones and the total distance of ambulatory movements. Anxiety is inversely related to the time spent in the central area and directly related to the thigmotaxis index. The significance of the difference was estimated by an analysis of variance (ANOVA) with Dunnett’s post hoc test (* *p* < 0.05 vs. C_12/12_, # *p* < 0.05 vs. DM_12/12_). For additional information, see Figure 2.

Anxiety-like behaviour in the OFT is associated with an increased time and ambulation distance in the peripheral fields of the test box. Representative images of rat ambulation in the OFT are presented in Figure 4A–D. The total and centre ambulation distance in OFT were significantly lower in DM_12/12_ vs. C_12/12_ (*p* < 0.05, Figure 4E,F). However, in the DM_6/18_ group, these two parameters were both significantly increased when compared to the DM_12/12_ group (*p* < 0.05; Figure 4E,F). Similarly, the time spent in the centre of the field and the number of rearings were also significantly lower in DM_12/12_ vs. C_12/12_ (*p* < 0.05, Figure 4G,K), with both parameters being significantly higher in DM_6/18_ vs. DM_12/12_ (*p* < 0.05; Figure 4G,K). These data suggest that according to the OFT, DM induced anxiety-like behaviour, while the shortening of the photoperiod exerted an anxiolytic effect.

Peripheral ambulation distance and the velocity in the periphery were also significantly lower in DM_12/12_ vs. C_12/12_ (*p* < 0.05, Figure 4H,J). On the other hand, these parameters were significantly higher in DM_6/18_ when compared to DM_12/12_ (*p* < 0.05, Figure 4H,J). The same trend between the groups was observed when the velocity in the central fields was analysed. While the velocity in the central field was significantly lower in DM_12/12_ vs. C_12/12_ (*p* < 0.05, Figure 4I), it was significantly higher in DM_6/18_ by comparison with the DM_12/12_ group (*p* < 0.05, Figure 4I). These results indicate that DM reduces exploratory behaviour of rats in EPM, while a shortened photoperiod reverses this effect. On the other side, there was no significant difference related to the thigmotaxis index between the observed groups (*p* > 0.05; Figure 4L).

### 3.3. Oxidative Stress

**Figure 5:**
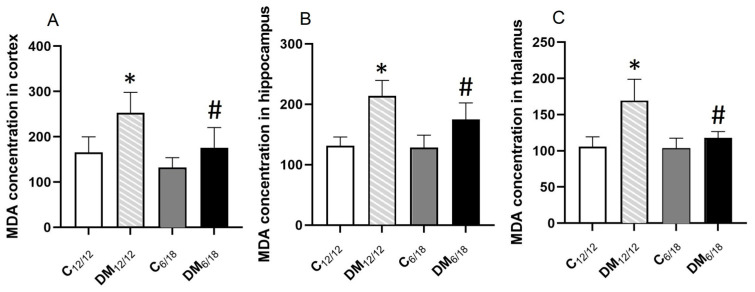
The effect of shortened daily photoperiod on the malondialdehyde (MDA) level in (**A**) cortex, (**B**) hippocampus and (**C**) thalamus. The significance of the difference was estimated by an analysis of variance (ANOVA) with Dunnett’s post hoc test (* *p* < 0.05 vs. C_12/12_, # *p* < 0.05 vs. DM_12/12_). For additional information, see Figure 2.

MDA concentration, a marker of lipid peroxidation, in the cortex was significantly higher in DM_12/12_ rats (257.44 ± 33.43 nmol/mg prot) when compared to the C_12/12_ group (166.50 ± 27.44 nmol/mg prot, *p* < 0.05). Conversely, the cortical MDA level was significantly lower in DM_6/18_ (169.50 ± 31.34 nmol/mg prot) by comparison with DM_12/12_ (*p* < 0.05; Figure 5A). Similar to the cortex, the MDA concentration in both the hippocampus and thalamus was significantly higher in DM_12/12_ in comparison to C_12/12_ (*p* < 0.05, Figure 5B,C); however, it was significantly lower in DM_6/18_ vs. DM_12/12_ (*p* < 0.05; Figure 5B,C). These results clearly indicate that a shortened photoperiod reduces lipid peroxidation in the brain of diabetic rats.

**Figure 6:**
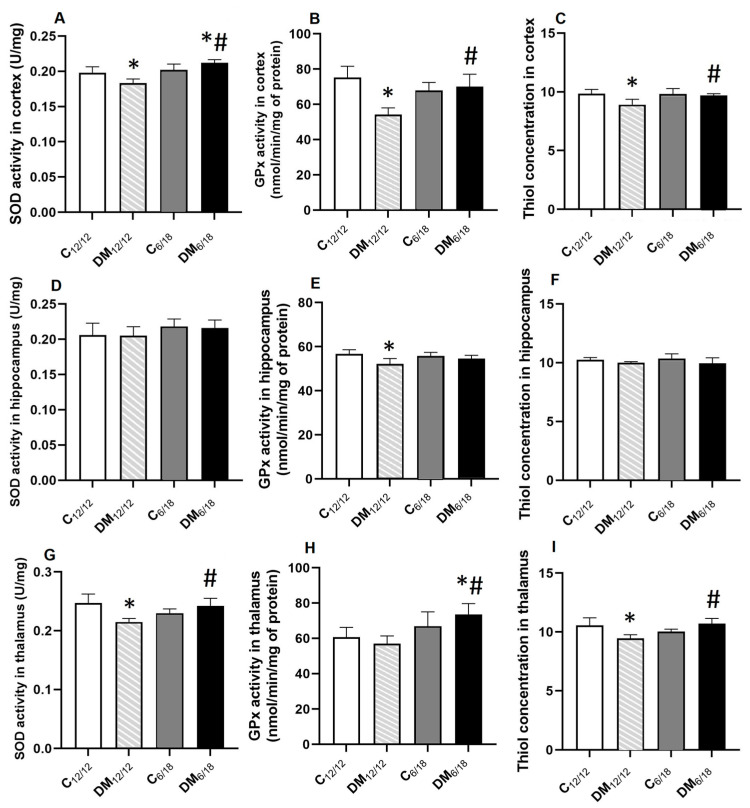
The activities of superoxide dismutase (SOD) (**A**–**C**) and glutathione peroxidase (GPx) (**D**–**F**) and the concentration of thiol groups (**G**–**I**) in the cortex, hippocampus and thalamus of diabetic rats exposed to a shortened daily photoperiod and a regular light/dark cycle. The significance of the difference was estimated by an analysis of variance (ANOVA) with Dunnett’s post hoc test (* *p* < 0.05 vs. C_12/12_, # *p* < 0.05 vs. DM_12/12_). For additional information, see Figure 2.

The activities of antioxidant enzymes and P-SH as an indicator of redox state are presented in Figure 6. The SOD, GPx activity and P-SH level in the cortex were significantly lower in DM_12/12_ vs. C_12/12_ (*p* < 0.05, Figure 6A–C). However, a shortened photoperiod reversed these changes and all previous parameters were significantly higher in DM_6/18_ in comparison to the DM_12/12_ group (*p* < 0.05; Figure 6A–C). SOD activity in the cortex was even significantly higher in the DM_6/18_ group (0.21 ± 0.01 U/mg prot) when compared with C_12/12_ (0.19 ± 0.01 U/mg prot; *p* < 0.05, Figure 6A).

In contrast, no significant difference in SOD activity or P-SH concentration was evident in the hippocampus between the control and experimental groups (*p* > 0.05; Figure 6D,F). Only GPx activity in the hippocampus was significantly lower in DM_12/12_ (52.14 ± 2.34 nmol/min/mg prot) vs. C_12/12_ (56.57 ± 1.84 nmol/min/mg prot; *p* < 0.05; Figure 6E) with no significant changes between DM_6/18_ (54.51 ± 1.55 nmol/min/mg prot) and DM_12/12_ (*p* > 0.05; Figure 6E).

In the thalamus, the SOD activity and P-SH concentration were significantly reduced in the DM_12/12_ group when compared with C_12/12_ (*p* < 0.05; Figure 6G,I). Both changes were reversed by a shortened photoperiod, and SOD activity and P-SH concentration were increased in DM_6/18_ in comparison to the DM_12/12_ group (*p* < 0.05). Interestingly, while the GPx activity was not different in DM_12/12_ (56.86 ± 3.89 nmol/min/mg prot) vs. C_12/12_ (61.12 ± 4.36 nmol/min/mg prot; *p* > 0.05), the activity of this enzyme in the thalamus was significantly higher in DM_6/18_ (73.12 ± 4.94 nmol/min/mg prot) when compared to both C_12/12_ (*p* > 0.05) and DM_12/12_ (*p* > 0.05; Figure 6H).

**Figure 7:**
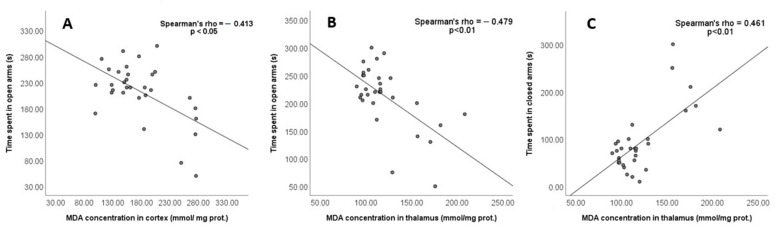
The correlation between MDA in brain structures and time spent in the open and closed arms of the EPM was analysed using Spearman’s test. The correlation was considered significant if *p* < 0.05. (**A**) Time spent in open arms and MDA concentration in cortex; (**B**) Time spent in open arms and MDA concentration in thalamus; (**C**) Time spent in closed arms and MDA concentration in thalamus.

A significant negative correlation was found between the time spent in the open arms of the EPM and the MDA concentration in the cortex (Spearmen rho = −0.413, *p* < 0.05; Figure 7A) and the thalamus (Spearman rho = −0.479, *p* < 0.01; Figure 7B), while there was no significant correlation between the time in the open arms and the MDA level in the hippocampus (Spearman rho = −0.417, *p* > 0.05). Conversely, the positive correlation between the time spent in the closed arms and the MDA level was found only in the thalamus (Spearman rho = 0.461, *p* < 0.01; Figure 7C).

**Figure 8:**
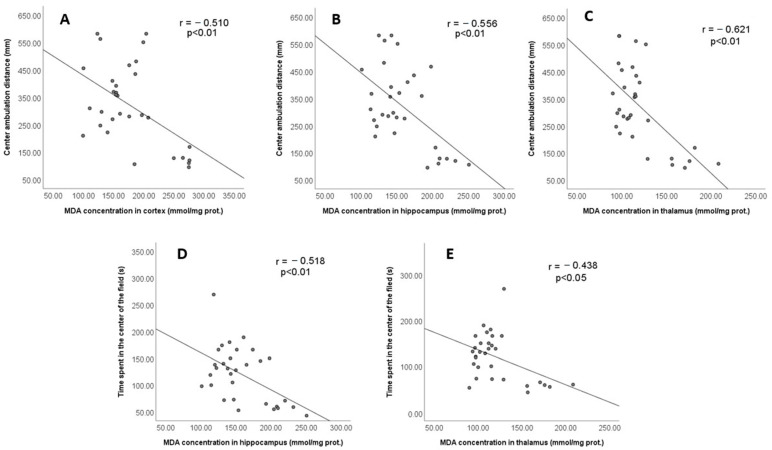
The correlation between MDA in brain structures and parameters of anxiety-like behaviour in the OFT was analysed using Pearson’s test. The correlation was considered significant if *p* < 0.05. (**A**) Center ambulation distance and MDA concentration in cortex; (**B**) Center ambulation distance and MDA concentration in hippocampus; (**C**) Center ambulation distance and MDA concentration in thalamus; (**D**) Time spent in the center of the field and MDA concentration in hippocampus; (**E**) Time spent in the center of the field and MDA concentration in thalamus.

A significant negative correlation was found between the centre ambulation distance in the OFT and the MDA concentration in the cortex (r = −0.510, *p* < 0.01; Figure 8A), hippocampus (r = −0.556, *p* < 0.01; Figure 8B) and thalamus (r = −0.621, *p* < 0,01; Figure 8C). Similarly, the time spent in the centre of the field also significantly negatively correlated with the MDA level in the hippocampus (r = −0.518, *p* < 0.01; Figure 8D) and the thalamus (r = −0.438, *p* < 0.05; Figure 8E). These data collectively indicate that the antioxidant effect of a shortened daily photoperiod correlates with its anti-anxiety effect in diabetic rats.

### 3.4. Lipid Status and FAMEs Concentration

Although the total serum cholesterol and HDL tend to change in diabetic rats, no significant alterations in these parameters were observed in DM_12/12_ vs. C_12/12_ group (*p* > 0.05). However, HDL cholesterol was significantly higher in DM_6/18_ group compared with both C_12/12_ and DM_12/12_ groups (*p* < 0.01). Triglyceride concentration was significantly higher in DM_12/12_ vs. C_12/12_ group (*p* < 0.01) and lower in DM_6/18_ in comparison with the DM_12/12_ group (*p* < 0.01; Table 1).

In the DM_12/12_ group, the vaccenic acid (C18:1ω7) concentration within serum lipids was significantly lower when compared with C_12/12_ group(*p* < 0.01). On the other hand, in DM_6/18_, the oleic (C18:1ω9), vaccenic (C18:1ω7), dihomo-γ-linolenic (C20:3) and DHA (C22:6) acid concentrations were significantly higher than in the DM_12/12_ group (*p* < 0.01). No significant changes in the concentrations of palmitic (C16:0), stearic (C18:0), palmitoleic (C16:1), γ-linolenic (C18:3ω6), linolelaidic (C18:2), arachidonic (C20:4), docosatetraenoic (C22:4), α-linoleic (C18:3ω3), EPA (C20:5) or DPA (C22:5) acids were observed (*p* > 0.05; Table 1).

**Figure 9:**
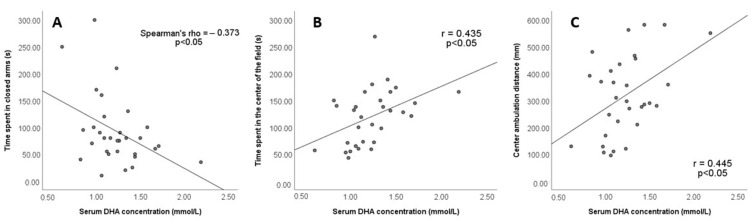
The correlation between FAME concentrations and anxiety parameters in EPM and OFT was analysed using Spearman’s test. The correlation was considered significant if *p* < 0.05. (**A**) Time spent in closed arms and serum DHA concentration; (**B**) Time spent in the center of the field and serum DHA concentration; (**C**) Center ambulation distance and serum DHA concentration.

The analyses of the correlation between DHA level and anxiety parameters are shown in Figure 9. Serum DHA concentration negatively correlates with the time spent in the closed arms of the EPM (Spearman rho = −0.373, *p* < 0.05; Figure 9A). Conversely, serum DHA concentration positively correlates with time spent in the central fields of the open-field arena (r = 0.435, *p* < 0.05; Figure 9B) and the centre ambulation distance (r = 0.445, *p* < 0.05; Figure 9C). No significant correlations were found between the serum concentrations of other fatty acids and anxiety parameters.

## 4. Discussion

Both clinical and animal studies have confirmed that diabetes-associated affective disorders are nowadays of wide concern apart from other diabetic complications [38,39]. Diabetes has been shown to be accompanied with neuroinflammation, oxidative stress and lipid peroxidation in the brain, which may contribute to neurodegeneration and impaired neuronal function leading to anxiety development [40,41]. Our results are in accordance with these findings, since the model of DM used in the present study was associated with anxiety-like behaviour and lipid peroxidation in the brain (Figure 3, Figure 4 and Figure 5).

Weight reduction, a reduced intake of saturated fat and physical activity are well known lifestyle measures for the prevention of DM and for the delay of diabetic complications, including anxiety. Our results clearly showed that a reduced light exposure may be an additional lifestyle measure that relieves diabetes-induced anxiety. It is generally accepted that at least two ethological tests (EPM, OFT and light/dark test) have to be included in the analysis, since a single test could reveal inconclusive results [31,42]. A shortened photoperiod reduced anxiety-like behaviour in both EPM and OFT. The anxiolytic effect of a shortened photoperiod in EPM was evident as a prolongation of the time spent in the open arms of the maze, as well as a reduced time spent in the closed arms (Figure 3). Similarly, OFT revealed that diabetic rats exposed to shortened photoperiod spent more time in the central area, although the thigmotaxis index was not changed by either diabetes or photoperiod shortening. Increased time spent in the central area has been generally accepted as an indicator of reduced anxiety in animals [42]. Total and peripheral ambulation distance, as well as the velocity, indicate reduced exploratory behaviour in diabetic rats, and all of these changes were reversed after photoperiod shortening. However, these parameters were decreased to a lesser extent than ambulation in the central zones, further confirming the relevance of time spent in the central area in the evaluation of anxiety-like behaviour. Additionally, diabetic rats did not have locomotor issues visible by inspection of their general behaviour (Figure 4). The combined interpretation of EPM and OFT results indicate that apart from increasing exploratory activity, the shortened photoperiod exerts an anxiolytic effect.

We propose that one mechanism contributing to the anxiolytic effect of the shortened photoperiod may be a reduction in oxidative stress. The first evidence linking oxidative stress and anxiety was indirect [42,43,44,45]. A sucrose-rich diet increased protein oxidation in the frontal cortex associated with anxiety-like behaviour [43]. A study in mice has shown that vitamin E deficiency causes anxiety associated with increased oxidative stress [44], while Berry et al. [45] have shown that aging is related to both anxiety-like behaviour and oxidative injury. Direct evidence of the role of oxidative stress in the development of anxiety was provided in a recent study [7] which shoed that edaravone ameliorates anxiety-like behaviour through a reduction in the MDA level and an increase in antioxidant enzymes in the hippocampus and prefrontal cortex. Similarly, we found in the present study that a shortened photoperiod significantly alleviated DM-induced lipid peroxidation in the cortex, hippocampus and thalamus (Figure 5). Our study also showed the correlation between the MDA level and anxiety-like behaviour in both the EPM and OFT, thus suggesting that the antioxidant effect of the shortened photoperiod may mediate its anti-anxiety effect.

However, the mechanisms of the antioxidant effect appear to be region specific. The shortened photoperiod increased the activity of SOD and GPx, as well as the concentration of thiol groups in the cortex and thalamus, but no changes were observed in the hippocampus (Figure 6). These regional differences were also found in our previous studies [46,47,48] and may be explained by different expressions of various antioxidant enzymes depending on the brain region. Antioxidant enzymes, such as SOD and GPx, display a higher activity in the brain than catalase [49]. There is also a different susceptibility of various brain regions to oxidative injury accompanied with differences in fatty acid contents [50].

The anxiolytic and antioxidant effects of reduced light exposure may be mediated by melatonin (Figure 2C). Clinical studies evaluating pre- and postoperative anxiety in patients have confirmed that melatonin given as a premedication reduces anxiety compared to a placebo [51]. Melatonin has been shown to alleviate depression in a rat model by reducing neuroinflammation via increased short-chain fatty acid production [52]. Conversely, melatonin alleviates oxidative stress by reducing MDA [53], by increasing GSH level and by the up-regulation of Nrf2—a transcription factor that induces the expression of antioxidant enzymes [54]. Similar to our study, Saxena et al. [55] have shown that melatonin administration reduces streptozotocin-induced lipid peroxidation in the rat hippocampus.

The reduction in anxiety caused by a shortened photoperiod also negatively correlates with an increased serum concentration of DHA (Figure 9, Table 1). The role of DHA in the development of anxiety has been previously described [56,57,58]. DHA deficiency in the brain due to transporter deficiency in endothelial cells of the brain microvessels results in severe neuronal loss in the hippocampus and cerebellum and the development of anxiety [56]. Human studies have revealed that the DHA level in plasma and red blood cell membrane is reduced in subjects with social anxiety disorders [59]. Direct evidence of the role of DHA deficiency in anxiety has been provided by interventional studies with DHA supplementation. DHA may be used as an adjuvant therapy in patients with anxiety resistant to the currently used drugs [57]. EPA/DHA administration was found to alleviate anxiety and depression symptoms in risperidone-treated psychotic patients [58]. Similarly, an animal study on obese rats reported decreased anxiety-like behaviour after DHA/EPA supplementation associated with a reduced plasma concentration of IL-6 and TNF-α, as well as suppressed TNF-α synthesis in the prefrontal cortex [60].

However, the present study does not provide the evidence of the causal relation between serum DHA level and anxiety-like behaviour. The increase in serum PUFA does not essentially reflect changes in the brain fatty acid profile, since a poor correlation has been found between saturated fatty acids, MUFA and PUFA in the brain and serum, except for α-linoleic acid [61]. Thus, the correlation between DHA concentration and anxiety-like behaviour found in our study suggests that the serum DHA increase may be used as a potential marker of the anxiolytic effect of a shortened daily photoperiod rather than pointing to a mechanistic role in the anxiety reduction.

The role of other changes in the fatty acid profile in the anti-anxiety effect of a shortened photoperiod is not completely clear. Although the serum concentrations of the MUFAs C18:1ω7 and C18:1ω9 were elevated after reduced exposure to light (Table 1), no correlation was found between anxiety-like behaviour and these fatty acid concentrations. This indicates that fatty acids except for DHA have no role in DM-induced anxiety nor in the protective effect of a shortened photoperiod. Previous studies have provided contradictory results on the role of C18:1ω7 and C18:1ω9 in the development of anxiety. While an Australian study has shown that anxiety risk rises with increased C18:1ω9 level [62], a recent cross-sectional study in Iranian women aged 18–49 has shown that an increased intake of oleic acid results in reduced anxiety [63]. There are no studies which have investigated the role of vaccenic acid in neuroinflammation, oxidative stress or behavioural disturbances, so its role in mediating effects of shortened photoperiod must be further clarified.

Diabetes caused hypertriglyceridemia in the present study, while it had no effect on the total and HDL cholesterol level (Table 1). However, a shortened daily photoperiod reduced serum triglyceride and increased serum HDL cholesterol concentrations in diabetic rats (Table 1), indicating that reduced exposure to light may have a protective effect against diabetes-induced dyslipidaemia. The role of lipid changes in the anxiolytic effect of a reduced daily photoperiod is still not clear. Interestingly, an increased HDL has been found to be associated with negative mood, while an increased triglyceride level improved mood in anxious patients [63].

A limitation of this study is the incompletely established translational potential in humans. The effect of lighting on behaviour has been found to be different in diverse species, strains and substrains. Circadian locomotor activity has been found to be increased in B6J compared with B6N mice, while anxiety-like behaviour is more prominent in B6N mice under basal conditions [64]. Conversely, the B6J substrain is more susceptible to circadian misalignment-induced behavioural changes which may be attributed to genetic differences [64]. This clearly suggests that the genetic background may influence the effect of a shortened photoperiod on anxiety, as well as on biochemical parameters. Additional limitations may be the nocturnal activity of rats, which substantially differs from humans. However, melatonin secretion in rats is also maximal during the night, and this enables the translation of melatonin effects to humans. However, the translational potential of this study should be further investigated in a human population.

## 5. Conclusions

Based on our results, it may be concluded that a shortened daily photoperiod alleviates anxiety-like behaviour in a rat model of streptozotocin-induced DM, partly through a decrease in lipid peroxidation. Mechanisms of antioxidant effects vary in a region-dependent manner with an increase in SOD, GPx activity and thiol groups concentration in the cortex and thalamus. Additionally, a shortened photoperiod increases the serum docosahexaenoic acid concentration that correlates with the reduction in anxiety-like behaviour. Docosahexaenoic acid may be used as a marker of anxiety-like behaviour in DM. This is the first study showing that a reduced exposure to light, including escape of social jet lag and reduced usage of cell phones and computers during the night, may be considered as a novel lifestyle measure for the alleviation of anxiety in the early stage of DM. However, the precise translational potential of this study must be further investigated in human populations.

## Figures and Tables

**Table 1:** Lipid status and fatty acid methyl ester concentrations in the serum of rats exposed to a shortened daily photoperiod and regular light/dark cycle.

Parameter [mmol/L]	C_12/12_	DM_12/12_	C_6/18_	DM_6/18_
Total cholesterol	1.04 ± 0.09	1.06 ± 0.06	1.08 ± 0.08	1.11 ± 0.10
TRG	0.65 ± 0.14	0.87 ± 0.17 **	0.66 ± 0.08	0.58 ± 0.12 ^##^
HDL	0.52 ± 0.05	0.45 ± 0.04	0.46 ± 0.03	0.63 ± 0.09 ** ^##^
C16:0	23.65 ± 1.55	22.83 ± 3.82	23.96 ± 2.91	23.62 ± 2.28
C18:0	12.36 ± 2.29	12.95 ± 1.41	11.76 ± 1.95	11.18 ± 2.05
C16:1	0.42 ± 0.06	0.42 ± 0.62	0.54 ± 0.09	0.53 ± 0.12
C18:1ω9	9.55 ± 2.74	8.34 ± 0.85	10.78 ± 1.41	12.16 ± 3.97 ^##^
C18:1ω7	2.04 ± 0.26	1.05 ± 0.27 **	1.81 ± 0.31	1.93 ± 0.62 ^##^
C18:2	28.97 ± 2.36	28.77 ± 4.07	26.57 ± 3.71	28.32 ± 3.07
C18:3ω6	0.12 ± 0.04	0.25 ± 0.12	0.15 ± 0.03	0.24 ± 0.18
C20:3	0.50 ± 0.08	0.44 ± 0.04	0.62 ± 0.17	0.66 ± 0.16 ^##^
C20:4	19.66 ± 2.07	19.87 ± 2.15	19.99 ± 2.73	20.68 ± 3.73
C22:4	0.43 ± 0.08	0.42 ± 0.07	0.39 ± 0.05	0.54 ± 0.15
C18:3ω3	0.32 ± 0.14	0.32 ± 0.11	0.28 ± 0.04	0.29 ± 0.08
C20:5	0.06 ± 0.03	0.09 ± 0.02	0.08 ± 0.05	0.07 ± 0.03
C22:5	0.45 ± 0.16	0.37 ± 0.07	0.35 ± 0.05	0.45 ± 0.13
C22:6	1.27 ± 0.31	0.99 ± 0.19	1.18 ± 0.15	1.45 ± 0.38 ^##^

All values are expressed as means ± SD. The significance of differences was estimated by an analysis of variance (ANOVA) with Dunnett’s post hoc test (** *p* < 0.01 vs. C_12/12_, ## *p* < 0.01 vs. DM_12/12_). Abbreviations: TRG, triglycerides; HDL, high-density lipoproteins; C16:0, palmitic acid; C18:0, stearic acid; C16:1, palmitoleic acid; C18:1ω9, oleic acid; C18:1ω7, vaccenic acid; C18:2, linolelaidic acid; C18:3ω6, γ-linolenic acid; C20:3, dihomo-γ-linolenic acid; C20:4, arachidonic acid; C22:4, docosatetraenoic acid; C18:3ω3, α-linoleic acid; C20:5, eicosapentaenoic acid; C22:5, docosapentaenoic acid; C22:6, docosahexaenoic acid.

## Data Availability

The data presented in this study are available on request from the corresponding author. The data are not publicly available due to privacy or ethical restrictions.
